# Supplementary material for: Myofibril orientation as a metric for characterizing heart disease
Source: Biophys J. 2022 Jan 12;121(4):565–74. doi: 10.1016/j.bpj.2022.01.009 (PMC8874025; doi:10.1016/j.bpj.2022.01.009)
Supplement: Document S1. Supporting methods, Figures S1 and S2, and Table S1 [file mmc1.pdf]

Biophysical Journal, Volume *121*

Supplemental information

Myofibril orientation as a metric for characterizing heart disease

Weikang Ma, Henry Gong, Vivek Jani, Kyoung Hwan Lee, Maicon Landim-Vieira, Maria Papadaki, Jose R. Pinto, M. Imran Aslam, Anthony Cammarato, and Thomas Irving

Supplemental Methods

X-ray Data Analysis. The data were analyzed using data reduction programs from the MuscleX software package developed at BioCAT (1). The angular divergence of the 1,0 equatorial X-ray reflections was measured by the "Scanning Diffraction" routine in the MuscleX software package. Briefly, the routine obtains 2D and 1D radially integrated intensities of the pattern. The 1D radially integrated intensity trace (red box in Fig S1) was fit assuming Gaussian profiles as a function of radial spacing for the diffraction peaks to calculate the standard deviation (width σ) of the peak intensity distribution in the radial direction. In this process, the routine obtains the integrated intensity of each equatorial reflection as a function of the integration angle (white box in Fig S1). Gaussian profiles (Fig 1D) are fit to the projected peak intensities to find the standard deviation of the orientation angle (angle σ) to calculate the angular divergence.

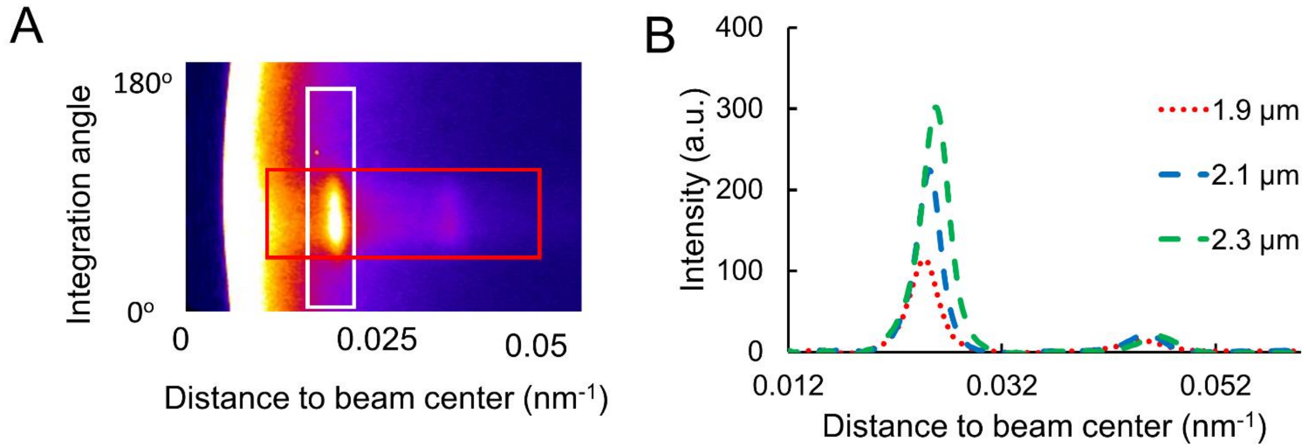

Figure S1. Determination of width σ: (**A**) Radially integrated X-ray pattern. White box: Integrates area of 1,0 reflection as a function of integration angle. Red box: Integrates area of 1,0 reflection as a function of the distance to beam center. (**B**) Radially integrated 1D intensity trace of 1,0 reflection (red box)

Sample Preparation for Electron Microscopy. Cardiac muscle fibers (< 2mm diameter) were excised from fresh or frozen pig heart ventricular wall and pinned down onto a Sylgard^{TM} substrate under slight tension. Fibers were fixed with 2.5% glutaraldehyde in 0.1M cacodylate buffer pH 7.2 for 2 hours at room temperature followed by continued fixation overnight at 4 °C. Samples were then rinsed in cacodylate buffer three times for 20 minutes each at 4°C. The fibers were shipped overnight on ice from to the University of Massachusetts Medical School. The fibers were cut into small pieces and post-fixed with1% w/v OsO_4 in distilled water, then dehydrated in a series of graded ethanol solutions and embedded in Epon. Sections (70 nm thick) were stained with uranyl acetate followed by lead citrate as described (1). The samples are examined with an FEI Tecnai G2 Spirit electron microscope at 120 kV. Images were collected on a 4k X 4k CMOS camera (Gatan Rio 9). The A-band, Z-line, and sarcomere length were measured using ImageJ software (NIH).

Image Analysis of Electron Micrographs. Surrogates for width and angle sigma measured by X-ray diffraction were determined from power spectra from low magnification (8200 x) electron microscopy images (Figure S2 A). Briefly, two dimensional fast Fourier transforms, and magnitudes were calculated to generate a 2D power spectrum (Figure S2 B). The resulting magnitude images were rearranged such that the zero-frequency component is positioned at the center of the array. The equator was manually determined, and the power spectra were rotated such that the equator was horizontal. The intensity corresponding to the (1,0) intensity was manually selected for analysis. Numerical radial and angular integrals were quantified for the selected analysis region with 0.01-degree resolution and 0.01-pixel resolution, respectively. Following numerical integration, profiles were normalized to have an integral of one and fit to a univariate Gaussian, and variances were acquired. Width sigma was the variance of the Gaussian fit to the radial integral profile, while angle sigma was the variance of the Gaussian fit to the angular integral profile. The algorithm described was implemented in MATLAB R2018b (Mathworks), with code made available from the authors on request. An example output of the code is shown in **Figure S2**. Finally, for each EM image, sarcomere length was determined using ImageJ from at least 20 sarcomeres in each image and the scale bars provided with the micrographs. The pixel-sarcomere length calibration was also used to quantify the distance from the center of the (1,0) intensity from the power spectra, which corresponds to inter-myofibrillar spacing.

Supplemental Results

Eight individual thin section electron micrographs from porcine myocardium samples with an average sarcomere length of ~2 µm and fifteen individual electron micrographs from samples with an average sarcomere length of ~2.3 µm, each containing an average of 13.3 (range 9-20) myofibrils, were selected and analyzed using the power spectrum analysis procedure as described in the supplemental methods. Figure S2 A shows one such electron micrograph from a thin section containing ~12 myofibrils. Note that the long axes of the myofibrils are roughly, but not perfectly parallel to each other so that the long axes of the myofibrils have a distribution of angles around the average long axis. It is the width of this distribution (Angle Sigma) that is meant by myofibrillar disorientation. Table S1 shows the results of the results of the analysis of the power spectra of the electron microscopy images as described in the supplemental methods. There is a significant decrease ($p = 0.005$) in angle sigma (myofibrillar disorientation) when sarcomere length increases from 2 to 2.3 µm, qualitatively and quantitatively similar to corroborating the results of the X-ray diffraction studies of mouse myocardium shown in Figure 2A. During stretch, the distance between adjacent myofibrils increased slightly but significantly ($p=0.049$) and the width of the distribution of these inter-myofibrillar spacings appeared to increase but this difference was not significant ($p=0.083$), possibly because of the noise in the data (Figure S2 D). Note that this measurement is not directly analogous to the width sigma measured by X-ray diffraction which is due, at least partly, to the width of the distribution of *inter-myofilament* lattice spacings.

In the X-ray diffraction measurements, the sample volume in the X-ray beam is ~250 x 250 x 250 µm or ~ 1.6×10^7 µm^3 and the sample is translated during the exposure by one to several mm increasing the sample volume to at ~ 6×10^7 µm^3 or more. In the electron micrographs, the sample volumes in each micrograph are 70 nm x 15 x 15 µm or 17 µm^3, many orders of magnitude less than the X-ray diffraction volume. it would, therefore, be highly impractical to

sample sufficient numbers of longitudinal EM sections to be statistically equivalent to the information contained in a single X-ray pattern. The sample volumes in the histological sections, on average 8. X 10^7 µm^3, are much larger than in the electron micrographs. However, individual myofibrils are not visible in these micrographs and the number of myocytes typically visible in a histological preparation are of the same order as the number of myofibrils visible in a thin section electron micrograph. Therefore, a similar number of independent structures (10-20) are being interrogated in individual sections with both the histological and electron microscopy measurements, much less than the many thousands of myofibrils interrogated in the X-ray measurements.

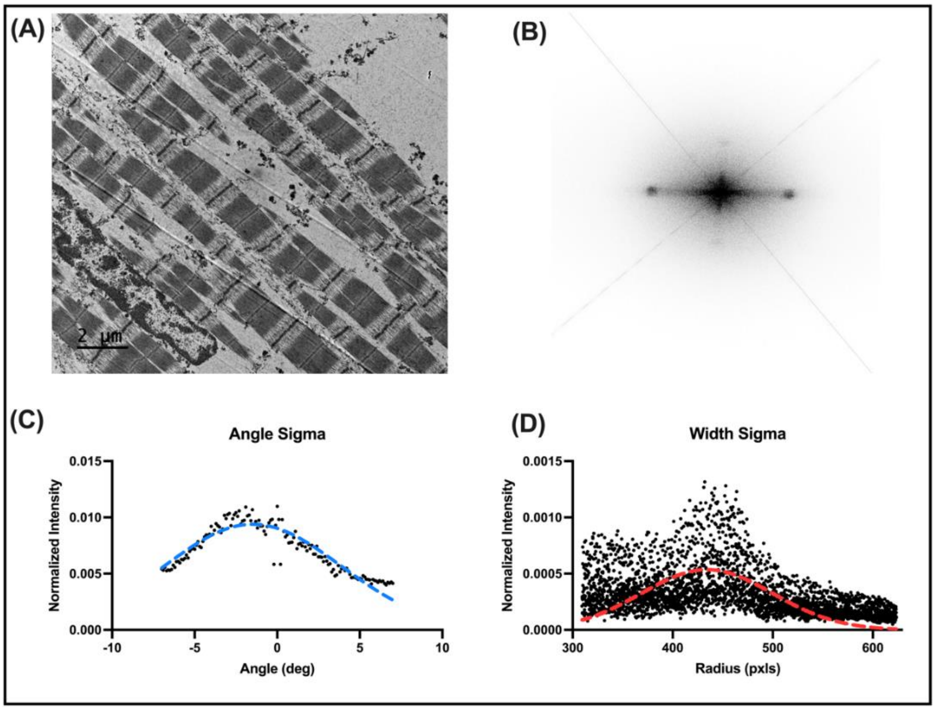

Figure S2. EM Image Analysis Workflow. (A) Representative EM image. (B) Power Spectrum. Spectrum was manually rotated to have the equator aligned horizontally. (C) Gaussian fit (blue) to quantify angle sigma of the angular integral of the (1,0) intensity from the power spectrum. (D) Gaussian fit (blue) to quantify width sigma from the radial integral of the (1,0) intensity from the power spectrum.

Table S1

**Sarcomere Length (µm)**	**Inter-myofibrillar Spacing (µm)**	**Angle Sigma (rad)**	**Width Sigma (µm)**
1.97 ± 0.012 (8)	1.70 ± 0.026 (8)	0.180 ± 0.038 (8)	0.276 ±0.044 (8)
2.30 ± 0.021 (15)	1.79 ± 0.024 (15)	0.142 ± 0.003 (15)	0.347 ±0.020 (15)

Supplemental References

1. Reynolds ES (1963) The use of lead citrate at high pH as an electron-opaque stain in electron microscopy. J Cell Biol 17:208-212
2. Jiratrakanvong, J., J. Shao, M. Menendez, X. Li, J. Li, W. Ma, G. Agam, and T. Irving. 2018. MuscleX: software suite for diffraction X-ray imaging V1.13.1. doi:10.5281/zenodo.1195050.
