# Supplementary material for: Myofibril orientation as a metric for characterizing heart disease
Source: Biophys J. 2022 Jan 12;121(4):565–74. doi: 10.1016/j.bpj.2022.01.009 (PMC8874025; doi:10.1016/j.bpj.2022.01.009)
Supplement: Document S2. Article plus supporting material [file mmc2.pdf]

Biophysical Journal
Article

Biophysical Society

Myofibril orientation as a metric for characterizing heart disease

Weikang Ma,[1,*] Henry Gong,[1] Vivek Jani,[2,3] Kyoung Hwan Lee,[4] Maicon Landim-Vieira,[5] Maria Papadaki,[6] Jose R. Pinto,[5] M. Imran Aslam,[3] Anthony Cammarato,[3,7] and Thomas Irving[1]

[1]BioCAT, Department of Biology, Illinois Institute of Technology, Chicago, Illinois; [2]Department of Biomedical Engineering, The Johns Hopkins School of Medicine, The Johns Hopkins University, Baltimore, Maryland; [3]Division of Cardiology, Department of Medicine, Johns Hopkins University School of Medicine, Baltimore, Maryland; [4]Division of Cell Biology and Imaging, Department of Radiology, University of Massachusetts Medical School, Worcester, Massachusetts; [5]Department of Biomedical Sciences, Florida State University, Tallahassee, Florida; [6]Department of Cell and Molecular Physiology, Loyola University Chicago, Chicago, Illinois; and [7]Department of Physiology, Johns Hopkins University School of Medicine, Baltimore, Maryland

ABSTRACT Myocyte disarray is a hallmark of many cardiac disorders. However, the relationship between alterations in the orientation of individual myofibrils and myofilaments to disease progression has been largely underexplored. This oversight has predominantly been because of a paucity of methods for objective and quantitative analysis. Here, we introduce a novel, less-biased approach to quantify myofibrillar and myofilament orientation in cardiac muscle under near-physiological conditions and demonstrate its superiority as compared with conventional histological assessments. Using small-angle x-ray diffraction, we first investigated changes in myofibrillar orientation at increasing sarcomere lengths in permeabilized, relaxed, wild-type mouse myocardium from the left ventricle by assessing the angular spread of the 1,0 equatorial reflection (angle σ). At a sarcomere length of 1.9 μm, the angle σ was 0.23 $\pm$ 0.01 rad, decreased to 0.19 $\pm$ 0.01 rad at a sarcomere length of 2.1 μm, and further decreased to 0.15 $\pm$ 0.01 rad at a sarcomere length of 2.3 μm ($p < 0.0001$). Angle σ was significantly larger in R403Q, a MYH7 hypertrophic cardiomyopathy model, porcine myocardium (0.24 $\pm$ 0.01 rad) compared with wild-type myocardium (0.14 $\pm$ 0.005 rad; $p < 0.0001$), as well as in human heart failure tissue (0.19 $\pm$ 0.006 rad) when compared with nonfailing samples (0.17 $\pm$ 0.007 rad; $p = 0.01$). These data indicate that diseased myocardium suffers from greater myofibrillar disorientation compared with healthy controls. Finally, we showed that conventional, histology-based analysis of disarray can be subject to user bias and/or sampling error and lead to false positives. Our method for directly assessing myofibrillar orientation avoids the artifacts introduced by conventional histological approaches that assess myocyte orientation and only indirectly evaluate myofibrillar orientation, and provides a precise and objective metric for phenotypically characterizing myocardium. The ability to obtain excellent x-ray diffraction patterns from frozen human myocardium provides a new tool for investigating structural anomalies associated with cardiac diseases.

SIGNIFICANCE We introduce a precise and quantitative approach to directly measure myofibrillar and myofilament orientation in cardiac muscle under near-physiological conditions as a novel tool for phenotypically characterizing striated muscle systems. We use this technique to demonstrate that myocardium from disease model organisms and failing human myocardium suffers from greater myofibrillar disorientation compared with healthy controls. We also demonstrate that excellent diffraction patterns can be obtained from frozen and thawed human myocardium. Given the ready availability of frozen human heart tissue in tissue banks, this capability opens up a large space of potential experiments relating sarcomere structure to dysfunction in cardiac disorders.

INTRODUCTION

A great deal of effort has gone into elucidating the molecular interactions involved in thick and thin filament-based regulatory mechanisms of force production by the sarcomeres of cardiac muscle, which, when perturbed, can trigger disease (1–6). Much less scrutiny, however, has been given to the very basic question of how myofibrillar- and myofilament-level alignment may be affected by discrete pathologies and how such changes could impact myocardial function. Classically, myocyte disarray, defined in this article as the degree to which adjacent myocytes are oriented either obliquely or perpendicularly to each other, is a hallmark of hypertrophic cardiomyopathy (HCM) (7,8).

Submitted October 1, 2021, and accepted for publication January 11, 2022.

*Correspondence: wma6@iit.edu

Editor: Samantha Harris.

https://doi.org/10.1016/j.bpj.2022.01.009

© 2022 Biophysical Society.

This is an open access article under the CC BY license (http://creativecommons.org/licenses/by/4.0/).

HCM is further characterized by increased myocardial wall thickness, hyperdynamic contractile properties, impaired energy balance, incomplete myocyte relaxation, and diastolic dysfunction (9,10). Although HCM can lead to heart failure (HF), determining HCM-associated HF incidence is problematic due to substantial etiological and clinical heterogeneity (11). Nonetheless, population studies have estimated that more than 50% of patients diagnosed with pathological concentric cardiac hypertrophy present HF symptoms (12).

HF with a reduced ejection fraction (HFrEF) is defined by an ejection fraction of 40% or less, comprises roughly 50% of HF cases, and is characterized by cardiomyocyte loss caused by ischemia, mutation, myocarditis, or valvular disease (13). However, electron microscopy analysis of myocardial tissue in a canine model of HF showed disrupted myofilament structure and loss of regular filament lattice structure that likely contributed to depressed force production (14). Unfortunately, microscopy-based methods require painstaking analysis of tissue in a conditional manner and cannot resolve other critical information such as cross-bridge formation and quantification of structural changes with variable calcium concentrations and is more subject to sample error given the amount of tissue analyzed. These pitfalls, among others, provide an impetus for developing additional approaches that allow for an accurate, unambiguous, and objective analysis of myofilament and myofibrillar disarray in both preclinical models of HF and human disease, particularly HFrEF.

Small-angle x-ray diffraction of cardiac tissue is a uniquely powerful technique to assess sarcomere structure under near-physiological conditions. The thick filaments in the sarcomeres of vertebrate muscle are packed into a hexagonal lattice with interdigitated thin filaments in the trigonal positions. The equatorial x-ray diffraction pattern (Fig. 1) arises from the projected density of the mass in the A-band of the sarcomere. The 1,0 equatorial reflection arises from thick filaments, while the 1,1 equatorial reflection arises from both thick and thin filaments. The ratio of the intensity of the 1,1 equatorial reflection to that of the 1,0 equatorial reflection ($I_{1,1}/I_{1,0}$) is a measure of the relative degree of association of myosin heads with thin filaments, both under resting conditions (1,15–17) and during contraction (16–20). The distance between the 1,0 equatorial reflection to the beam center can be used to calculate the interfilament lattice spacing, d_{10}. There is more information in the equatorial pattern, however, in the form of the angular spread of the equatorial reflections relative to the equatorial axis. In striated muscle, the equatorial reflections are perpendicular to the myofibrillar longitudinal axis; thus, the angular spread of the equatorial reflections across the equator (Fig. 1) will be a direct measure of the degree of alignment of the sarcomeres relative to the long axis of the preparation. In this article, we will refer to the degree of departure from perfect angular alignment of myofibrils with the long axis of the preparation as "myofibrillar disorientation." The angular spread of x-ray reflections has previously been used to characterize the relative orientation of collagen fibers in connective tissue (21,22). However, this approach has not previously been used, to our knowledge, to assess the alignment of contractile machinery in cardiac tissue.

In this study, we first investigated the changes in myofibril structure with increased sarcomere length in mouse myocardium with the motivation of evaluating a fundamental sarcomere geometric mechanism that could help explain myofilament length-dependent activation (LDA), the phenomenon where increasing sarcomere length results in additional force for the same calcium concentration. Indeed, we observed improved myofibril alignment with increased sarcomere length in normal mouse myocardium, consistent with LDA. This observation prompted us to attempt to evaluate our technique's ability to quantify myofibril-level disarray and correlate this to cardiac disease. As a first step toward this goal, we studied myofibrillar disorientation

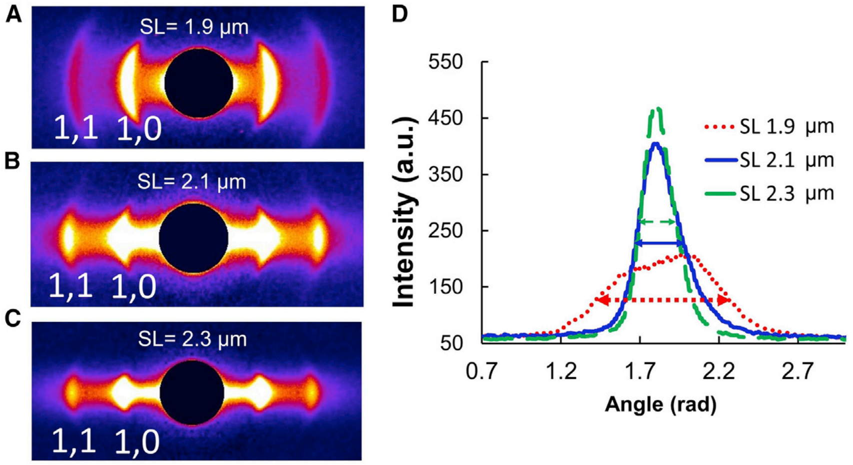

FIGURE 1 Equatorial x-ray diffraction patterns from permeabilized murine myocardium. Representative equatorial x-ray diffraction patterns from permeabilized mouse myocardium (*A–C*) and the angular intensity profile of the 1,0 equatorial reflection (*D*) at different sarcomere lengths. The full width at half maximum (~2.36 σ) of the peaks is indicated by the double-headed arrows at corresponding colors.

in transgenic porcine myocardium expressing R403Q mutant myosin. The R403Q mutation was the first identified HCM-causing mutation in β-cardiac myosin (23), and hearts from R403Q transgenic mice showed increased myocyte disarray using histological techniques (24). By measuring the angular spread of the equatorial reflections, we quantitatively confirmed that, in large-animal models, R403Q myocardium also suffers from a higher degree of myofibrillar disorientation as compared with control. We then extended our studies to human tissue, demonstrating for the first time, to our knowledge, that high-quality two-dimensional (2D) x-ray diffraction patterns, suitable for detailed structural analysis, can be obtained from frozen human heart tissue. Myofibrillar disorientation was quantified in right ventricular myocardium from nonfailing donors (Non-Failing) versus patients with HFrEF that was not preceded by HCM. We show that the HFrEF tissue displayed significant myofilament- and myofibril-level disorientation relative to Non-Failing controls.

MATERIALS AND METHODS

Mouse myocardium preparations

All procedures using live mice were done in accordance with protocols approved by the Institutional Animal Care and Use Committees of the Florida State University and the Illinois Institute of Technology. Wild-type mice ($n = 8$) were euthanized by deep anesthesia with isoflurane followed by cervical dislocation. Left ventricle papillary muscles were dissected from mice and permeabilized with 1% Triton X-100 in relaxing solution (containing 6.3 mM Na_2ATP, 6.48 mM $MgCl_2$, 10 mM EGTA, 100 mM N,N-bis(2-hydroxyethyl)-2-aminoethanesulfonic acid (BES), 10 mM phosphocreatine, 49.76 mM potassium propionate, 10 mM dithiothreitol (DTT), and creatine kinase 10 units/ml) overnight. The muscles were washed with fresh cold relaxing solution, and muscles were further dissected into fiber strips, clipped on aluminum T-clips, and stored in cold relaxing solution for the day's experiments.

Porcine myocardium preparation

The data from the porcine myocardium (samples taken from one heart each for both wild-type [WT] and R403Q) reported in this article were from a reanalysis of the patterns reported by Anderson et al. (25).

Human myocardium preparation

Human subjects were enrolled under protocols approved by the Institutional Review Boards at the Johns Hopkins University, the University of Pennsylvania, and the Gift-of-Life Donor Program of Pennsylvania (26). Human myocardial tissue was procured as described previously (26). In brief, failing hearts ($n = 5$) were obtained via explantation at the time of orthotopic heart transplantation, while nonfailing hearts ($n = 5$) were obtained from brain-dead organ donors. Nonfailing hearts were not used for transplantation because of donor age. Hearts were rapidly arrested using high-potassium cold-cardioplegia and open-chest surgical excision, then transported in cold Krebs-Henseleit buffer on wet ice to an on-campus laboratory. Tissue was then rapidly dissected, snap frozen in liquid nitrogen, and stored at −80°C until transport. Right ventricular (RV) septal tissue from patients showing low RV maximum isometric force (Fmax) (27,28) was used in all RV studies.

Frozen human RV tissues were permeabilized as previously described for porcine myocardium (25). In brief, pieces of frozen human RV tissues (3 mm^3) were put in relaxing solution (containing in mM: 100 BES, 10 EGTA, 6.6 $MgCl_2$, 10 creatine phosphate, 15 creatine kinase, 6.2 ATP, 5 NaN_3 [pH 7.0]) containing 1% Triton X-100 at room temperature for 2–3 h. The skinning solution was changed every hour. The muscle was then washed with relaxing solution before application of aluminum T-clips and stored in cold relaxing solution for the day's experiments.

Histology preparation and measurement of myocyte alignment

Conventionally prepared hematoxylin and eosin and Masson's trichrome-stained tissues were obtained from both nonfailing ($n = 4$) and failing human ($n = 4$) RV septal myocardium. Myofibrillar disarray was quantified using an image processing-based approach, described in detail elsewhere (24,29). In brief, between 4 and 6 cropped 1000 × 1000-pixel sections (~250 μm × 250 μm) of longitudinally sectioned RV myocardium at ×20 magnification were identified for analysis from both hematoxylin and eosin- and Masson's trichrome-stained sections for each patient. A longitudinally sectioned region was considered suitable for analysis if there was minimal fibrosis, few histological artifacts, and minimal spaces/cracks within the tissue. Identified regions were exported as ".png" images and imported into MATLAB (version R2018b, 2020; The MathWorks, Natick, MA). A 2D fast Fourier transform was applied to each image, and a power spectral density was determined as described previously (29). Next, for each angle, a histogram of the distribution of the relative number of myocytes aligned at each angle θ was determined, and the percent myocyte alignment, quantified as the relative proportion of myocytes within 20° of the mean myocyte alignment angle, was determined for each image.

Small-angle X-diffraction

Equatorial x-ray diffraction patterns were collected from freshly permeabilized muscle strips using the small-angle instrument on the BioCAT beamline 18ID at the Advanced Photon Source, Argonne National Laboratory (30). The x-ray beam was focused to ~0.06 × 0.15 mm at the detector plane. The sample-to-detector distance was ~3.5 m, and the x-ray wavelength was 0.103 nm. Isolated cardiomyocyte bundles (~200 μm in diameter, 2–3 mm long) were mounted between a force transducer (Model 402A; Aurora Scientific, Aurora, ON, Canada) and a static hook. Force was monitored using Muscle Dynamic Control system (Model 610A; Aurora Scientific). Sarcomere length was adjusted by laser diffraction using a 4-mW HeNe laser. Diffraction patterns were collected at sarcomere lengths of 1.9, 2.1, and 2.3 μm for mouse myocardium experiments and 2.0 μm for human myocardium experiments. X-ray exposures were 1 s at an incident flux of $\sim 3 \times 10^{12}$ photons/s, and the patterns were collected on a charge-coupled device-based x-ray detector (Mar 165; Rayonix, Evanston, IL).

X-ray data analysis

The data were analyzed using data reduction programs from the MuscleX software package developed at BioCAT (31). The angular divergence of the 1,0 equatorial x-ray reflections was measured by the "Scanning Diffraction" routine in the MuscleX software package. In brief, the routine obtains 2D and 1D radially integrated intensities of the pattern. The 1D radially integrated intensity trace was fit assuming Gaussian profiles as a function of radial spacing for the diffraction peaks to calculate the standard deviation (width σ) of the peak intensity distribution in the radial direction as shown in Fig. S1 *A*. In this process, the routine obtains the integrated intensity of each equatorial reflection as a function of the integration angle as illustrated in Fig. 1 *D*. Gaussian profiles are fit to the projected peak intensities to find the standard deviation of the orientation angle (angle σ) as a measure of the

angular divergence of the angle that the sarcomeres in the myofibrils make to the long axes of the preparation.

Statistics

All statistical comparisons made in this study use the number of samples that do not necessarily come from different individual subjects. The purpose of this study is to demonstrate the possible utility of the new technique, not to fully characterize any given preparation or pathology where the distinction between biological replicates (number of subjects) and technical replicates (number of samples interrogated) would be important. Statistical analyses were performed using GraphPad Prism 9 (Graphpad Software). The results are given as mean ± SEM unless otherwise stated. One-way paired ANOVA with Geisser–Greenhouse correction and Tukey multiple comparison test were used to compare columns in Fig. 2 in a pairwise manner. Two-tailed unpaired Mann–Whitney tests were used for the data from porcine and human myocardium shown in Figs. 3 and 4 ($^{ns}p \geq 0.05$, $^{*}p < 0.05$, $^{**}p < 0.01$, $^{***}p < 0.001$, $^{****}p < 0.0001$).

RESULTS

To establish a new approach for quantifying the degree of myofilament order, and how it changes with sarcomere length, in native myocardium, we first collected x-ray diffraction patterns from permeabilized WT mouse left ventricle papillary muscle at sarcomere lengths of 1.9, 2.1, and 2.3 μm under relaxing conditions. The equatorial 1,0 and 1,1 reflections were present at all three lengths. The intensity profiles, however, became better defined with increasing muscle sarcomere length, as shown in representative x-ray patterns (Fig. 1 *A–C*). The equatorial 1,0 and 1,1 reflections were visibly arced at a sarcomere length of 1.9 μm. The azimuthal angular width of the reflections became smaller as the sarcomere length increased, as shown by the angular integrated intensity profiles of the 1,0 reflections in Fig. 1 *D*. The standard deviation of the angular projection of the peak (angle σ) was plotted as a function of sarcomere length (Fig. 2 *A*), which showed that angle σ decreased by about 20% for every 10% increase in sarcomere length. At a sarcomere length of 1.9 μm, the angle σ for the 1,0 reflection was 0.23 ± 0.01 rad, which decreased to 0.19 ± 0.01 rad at a sarcomere length of 2.1 μm ($p = 0.005$) and further decreased to 0.15 ± 0.01 rad at a sarcomere length of 2.3 μm ($p < 0.0001$). To provide an easily visualized analogy for what is being measured as angle sigma by the x-ray diffraction experiments, we analyzed a dataset of thin-section electron micrographs from porcine myocardium at short and long sarcomere lengths, as described in the supporting material, with the results shown in Table S1. Angle sigma measured from the electron micrographs was 0.180 ± 0.038 rad ($n = 8$) at a sarcomere length of 1.97 μm decreasing to 0.142 ± 0.003 rad (15) at a sarcomere length of 2.3 μm, very similar to the results from x-ray diffraction of mouse myocardium, corroborating these findings.

The standard deviation of the radial width of the equatorial peaks (width σ) was also calculated as a function of sarcomere length. Width σ for the 1,0 reflection, a measure of heterogeneity in lattice spacing between myofibrils, remained unchanged when the sarcomere length was increased from 1.9 μm (1.40 ± 0.04 [10^{-3} nm^{-1}]) to 2.1 μm (1.41 ± 0.04 [10^{-3} nm^{-1}]; $p = 0.78$). However, width σ significantly increased at a sarcomere length of 2.3 μm (1.50 ± 0.05 [10^{-3} nm^{-1}]) when compared with sarcomere lengths of 1.9 μm ($p = 0.04$) and 2.1 μm ($p = 0.04$). These data demonstrate that the decrease in angle σ at longer sarcomere length is accompanied by a small but significant increase in lattice spacing heterogeneity in skinned, WT myocardium.

Our results from normal mouse myocardium raised the question whether these assays of myofilament ordering could be used to characterize myocardium from disease models. Therefore, we analyzed myocardium from R403Q porcine HCM model pig as a large-animal model expressing the well-characterized, HCM-causing R403Q myosin mutation. Myofibrillar orientation was assessed in permeabilized WT and R403Q porcine myocardium at a sarcomere length of 2.0 μm (Fig. 3). Qualitatively, the equatorial reflections are arced in R403Q myocardium, while the equatorial reflections are well-defined spots in WT myocardium (Fig. 3 *A*). Angle σ was significantly higher in R403Q myocardium (0.24 ± 0.01 rad) relative to WT myocardium (0.14 ± 0.005 rad) ($p < 0.0001$) (Fig. 3 *B*). In addition, width σ for the 1,0 reflection is significantly larger in WT myocardium (2.14 ± 0.04 [10^{-3} nm^{-1}]) than in R403Q myocardium (1.76 ± 0.06 [10^{-3} nm^{-1}]) ($p < 0.0001$) (Fig. 3 *C*). These data show the same trend as that from

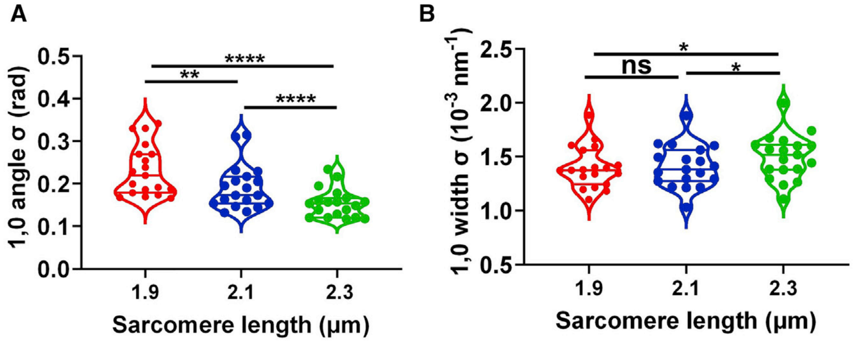

FIGURE 2 1,0 equatorial reflections in permeabilized mouse myocardium at different sarcomere lengths. (*A*) The angular standard deviation of 1,0 equatorial reflections (angle σ) from permeabilized mouse myocardium as a function of sarcomere length. (*B*) The standard deviation of the 1,0 equatorial reflections in radial direction (width σ) from permeabilized mouse myocardium as a function of sarcomere length ($^{ns}p \geq 0.05$, $^{*}p < 0.05$, $^{**}p < 0.01$, $^{****}p < 0.0001$).

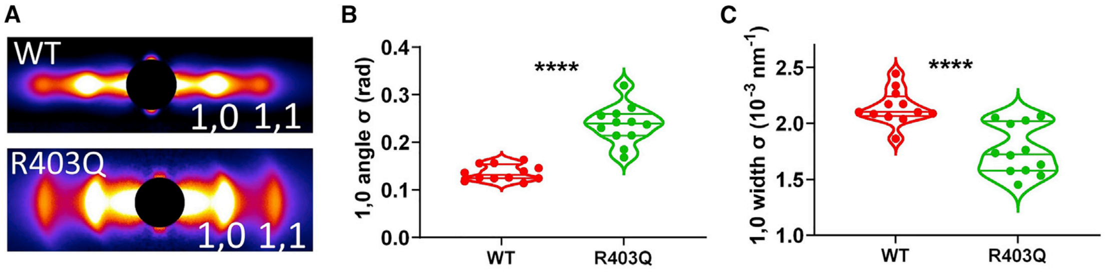

FIGURE 3 Myofibrillar orientation from permeabilized porcine myocardium. (*A*) Representative equatorial x-ray diffraction patterns from permeabilized WT and R403Q porcine myocardium. (*B*) The angular standard deviation of 1,0 equatorial reflections (angle σ) from permeabilized WT and R403Q porcine myocardium. (*C*) The standard deviation of the 1,0 equatorial reflections in radial direction (width σ) from permeabilized WT and R403Q porcine myocardium (****$p < 0.0001$).

previous studies using small rodent models of R403Q myosin-dependent HCM, which showed disarray at the myocyte level (24).

We next examined the possibility of applying our methods to phenotypically characterize myofibrillar and myofilament alignment in normal and diseased human myocardium. If x-ray diffraction patterns could be obtained from cryofrozen human heart tissue, they could offer a uniquely powerful and transformative tool for examining structure–function relationships in clinically relevant specimens. Fig. 4 shows that the structural integrity of the sarcomeres in these preparations was well preserved. In addition to the equatorial reflections addressed in this study, meridional reflections arising from the axial repeats of myofilaments are strong and extend beyond the sixth order myosin-based meridional reflection (M6). Layer lines arising from the quasi-helically ordered array of myosin heads around the thick filament backbone in relaxed muscle are clearly visible. The first myosin layer line is particularly strong.

To investigate myofilament disarray in human HFrEF, we studied the angular and radial profiles of 1,0 equatorial reflection from frozen healthy human myocardium and myocardium from patients with HFrEF under relaxing conditions at a sarcomere length of 2.0 μm. Specifically, we chose human HFrEF samples with depressed maximum calcium-activated force from RV permeabilized myocytes as described previously (27). The angle σ was significantly higher in HFrEF myocardium (0.19 ± 0.006 rad) than Non-Failing myocardium (0.17 ± 0.007 rad) ($p = 0.01$) (Fig. 5 *B*), indicating a higher degree of myofibrillar disorientation in HFrEF myocardium. The width σ for the 1,0 reflection was significantly higher in HFrEF myocardium (1.35 ± 0.03 [10^{-3} nm^{-1}]) than in Non-Failing myocardium (1.25 ± 0.03 [10^{-3} nm^{-1}]) ($p = 0.04$) (Fig. 5 *C*), demonstrating a greater degree in dispersion in lattice spacing between myofibrils in HFrEF myocardium.

Finally, we benchmarked our method for assessing myofibrillar orientation using x-ray diffraction against existing approaches that assess only myocyte orientation, which will be, at best, indirectly related to myobrillar/myofilament alignment. For a good example of a currently used protocol using histology to assess myocyte disarray, see Anderson et al. (25).

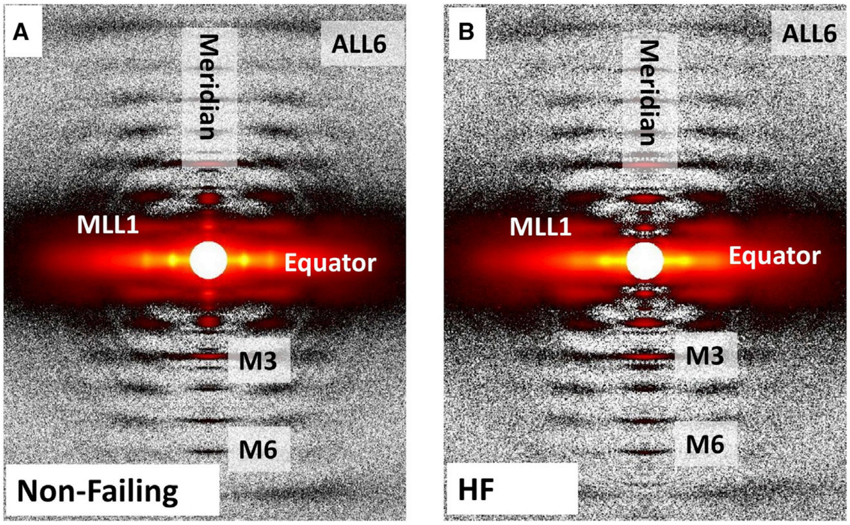

FIGURE 4 Representative x-ray patterns from frozen Non-Failing (*A*) and patients with HF (*B*) human myocardium. The 1,0 and 1,1 equatorial reflections, the third (M3) and sixth (M6) order myosin-based meridional reflections, first-order myosin-based layer lines (MLL1), and sixth order actin-based layer lines (ALL6) are as labeled.

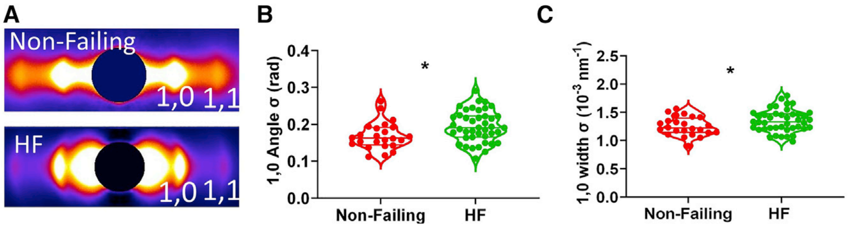

FIGURE 5 Human myocardium myofibrillar orientation by x-ray from Non-Failing and patients with HF. (*A*) Representative equatorial x-ray diffraction patterns from Non-Failing myocardium and HF myocardium. (*B*) The angular standard deviation of 1,0 equatorial reflections (angle σ) from permeabilized Non-Failing and HF human myocardium. (*C*) The standard deviation of the 1,0 equatorial reflections in radial direction (width σ) from permeabilized Non-Failing and HF human myocardium (*$p < 0.05$).

Representative Masson's trichrome sections from the same Non-Failing and HFrEF RV samples used for x-ray analysis are shown in Fig. 6 *A* and *B*, respectively. Quantification (see Materials and methods) of the percentage of aligned myocytes (Fig. 6 *D*) indicated that the percentage of aligned myocytes was significantly reduced in RV HFrEF histological sections compared with Non-Failing RV histological sections. Importantly, these myocyte alignment results are consistent with our results showing the degree of myofibrillar alignment ("myofibrillar disorientation") by x-ray diffraction (Fig. 5). It needs to be emphasized, however, that the two techniques do not measure the same thing, although they can be expected to be strongly correlated. The histology images in Fig. 6 *A* and *B* show only myocyte orientation. Myofibrils and sarcomeres are not visible. In contrast, x-ray diffraction is sensitive to structures (the sarcomeres) only within the myofibrils.

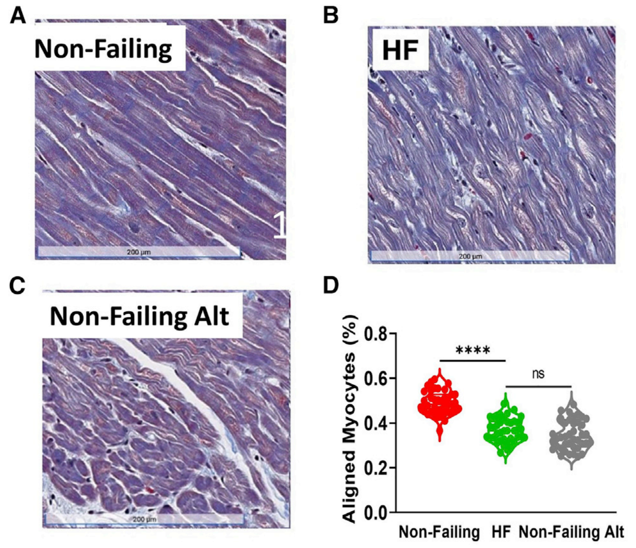

FIGURE 6 Human cardiomyocyte alignment by histology analysis from Non-Failing and patients with HF (HFrEF). Representative histology images from Non-Failing myocardium (*A*) and HF myocardium (*B*). (*C*) Representative histology images from Non-Failing myocardium at an alternative region. (*D*) The percentage of aligned myocytes in Non-Failing myocardium, HFrEF myocardium, and Non-Failing myocardium at alternative regions (Non-Failing Alt) that were not considered longitudinally aligned or suitable for analysis from histology analysis ($^{ns}p \geq 0.05$, ****$p < 0.0001$).

Next, to demonstrate the sensitivity of histological methods to user-based selection criteria, we calculated myocyte alignment in regions not considered suitable for analysis in nonfailing histological sections (denoted Non-Failing alt, Fig. 6 *C*). This analysis revealed that the percentage of aligned myocytes in these regions was similar to those from HFrEF histological sections (Fig. 6 *D*), suggesting that user criteria for histology analysis may be subject to bias and susceptible to false positives.

DISCUSSION

Characterization of myofibrillar orientation in permeabilized murine and porcine myocardium

The degree of myocyte alignment (or its converse, disarray) is commonly used to characterize healthy versus diseased heart tissue; however, the orientation of myofibrils and degree of disorder in myofilaments have been overlooked, largely because of the lack of available methods to assess these quantities.

In this study, we quantitatively showed, using x-ray diffraction, that the myofibrils in mouse cardiac muscle cells are relatively poorly aligned ("myofibrillar disorientation") at a sarcomere length of 1.9 μm with an angular divergence (angle σ) of 0.24 ± 0.02 rad, and that angular divergence significantly decreased to 0.15 ± 0.01 rad at a sarcomere length of 2.3 μm (Fig. 2 *A*). At longer sarcomere lengths, the decrease in angle σ indicates that the myofibrils in cardiac muscle cells are better aligned with the longitudinal axis. When myofibrils contract and produce force, the active force can be separated into both a radial component and an axial component relative to the long axis of the muscle preparation. Better aligned myofibrils in the longitudinal axis could lead to more axial force at longer sarcomere length by redistributing radial components of force to the axial direction and provide a partial explanation for the increased force in LDA. One other expected effect of myofibrillar disorientation would be to effectively increase the series elastic component of the muscle to require more muscle stretch before sarcomere length starts to increase.

The radial width σ of the 1,0 equatorial reflection did not change when the myocardium was stretched from 1.9 to 2.1 μm. A significant increase in width σ, however, was

observed when the sarcomere length was increased to 2.3 μm. The radial width σ in resting muscle is dominated by interfilament spacing heterogeneity (32) but can also include contributions from any other deviations from a perfect hexagonal lattice in the sarcomeres. Collectively, we call these effects "myofilament disarray." Interfilament spacing heterogeneity could come from the spread of lattice spacings among individual myofibrils (intermyofibrillar) and/or within individual myofibrils (intramyofibrillar) in the myocytes. An increase in the intramyofibrillar component of myofilament disarray at longer sarcomere length is most likely due to the increase in titin-based passive tension with increasing sarcomere length, being relatively modest between 2.0 and 2.1 μm and increasing exponentially at 2.3 μm and longer sarcomere lengths (33). Titin-based passive tension will have both radial and longitudinal components, which may vary between sarcomeres, but with an overall trend to compress the lattice and shorten the sarcomere (34). An increase in intermyofibrillar heterogeneity at longer sarcomere length is likely due to the nonhomogeneous transmission of passive force by cytoskeletal components within myocytes, such as the desmin intermediate filament network surrounding the myofibrils and interconnecting the Z-lines (35–37) and the costameres connecting the Z-lines to the sarcolemma (38,39). Interestingly, the expression levels of desmin in the heart have been shown to increase in HCM/diastolic dysfunction mouse models (40) and decrease in dilated cardiomyopathy (DCM) models (41). The sensitivity of both angle σ and width σ to sarcomere length emphasizes the need for the x-ray diffraction measurements to be done at a known, defined sarcomere length. Another consideration is that sarcomere length varies transmurally and may differ among the various cardiomyopathies, so it should be selected to be appropriate for the comparisons being made.

Remodeling of myocyte morphology is a complex process and is regulated by mechanical, hormonal, and hemodynamic stimuli (42). Pathological remodeling of cardiomyocytes is widely observed in end-stage HF (43,44). One of the end results of pathological heart remodeling is cardiomyocyte disarray, which is characteristic of cardiomyopathies, including HCM. Myocyte disarray has been proposed to be one of the criteria for the diagnosis of HCM (45,46). In this article, we extend this concept to the myofibrillar level by examining the degree of myofibrillar disorientation in transgenic animal models that are known to display myocyte disarray. Specifically, our results showed that in a porcine model of HCM, with the R403Q mutation, the myocardium displayed a greater than 70% increase in angle σ compared with WT myocardium. Interestingly, width σ (radial) was significantly smaller in R403Q myocardium compared with WT myocardium (Fig. 3 *C*), indicating a lower degree of interfilament heterogeneity. Fig. 2 *B* shows that radial width σ and angular σ of the 1,0 reflections are not necessarily coupled, and as such, they can be considered two independent parameters to characterize a given myocardial system.

Study of frozen human myocardium using x-ray diffraction

In this study, we used the methodology introduced in our studies of murine and porcine myocardium to examine the degree of myofibrillar disorientation from failing versus nonfailing human myocardium. Specifically, we investigated frozen explanted heart tissue from a subpopulation of patients with HFrEF with known RV myocyte dysfunction, as assessed by depressed maximum calcium-activated tension. Our results demonstrated that myocardium from patients with HFrEF displayed larger values of 1,0 angular σ compared with that from Non-Failing and suggests that myofibrillar disarray may contribute to both RV myocyte dysfunction and global RV dysfunction.

The mechanism of depressed sarcomere function in HFrEF as measured by maximal calcium activated tension has been explored in previous studies (27,28). For example, myofilament disarray has been shown to be responsible for diminished force production in a canine model of HF (14). Currently, however, the exact mechanisms by which myofilament disarray results in depressed force output are not known and may not be the only mechanism acting in a given situation. Now that we have a tool to quantify myofilament orientation, experiments can be designed to determine the extent to which myofilament-level disarray plays a role in the etiology of any myocardial disorder.

We also show for the first time, to our knowledge, that frozen human myocardium produces high-quality 2D x-ray patterns that may provide valuable information concerning not only the molecular structural basis of the functional behavior in healthy hearts but also can potentially resolve structural aberrations/correlates that may contribute to depressed or enhanced contractility commonly observed in cardiomyopathies. It has been demonstrated that myosin heads in resting muscle are distributed between the super relaxed state (SRX), characterized by a very low intrinsic ATPase rate, and the disordered relaxed state (DRX) where the intrinsic ATPase rates are much faster (47). The myosin heads in the DRX state are able to participate in cross-bridge formation during contraction, while myosin heads in the SRX state are unable to interact with actin but can be recruited by inotropic effectors, including sarcomere stretch, effectively serving as a reserve (48). The equilibrium between myosin heads in SRX and DRX state are important in balancing cardiac physiological functions, such as LDA (47). It has been proposed that many cardiomyopathies are caused by disruption of SRX and DRX equilibria and transitions between these states (49). X-ray diffraction is very sensitive to global structural transitions of myosin heads between SRX and DRX in the context of thick filament-based regulatory mechanisms as shown in studies in rodent and

porcine myocardium (3,25,50). The ability to obtain this kind of information, in addition to myofibrillar orientation, from human myocardium greatly enhances the value of this approach for translational studies as demonstrated in a preliminary report (51). A full report demonstrating increased myofibrillar disarray in myocardium from patients with HFrEF will appear elsewhere.

Comparison to histological measurements

In this article, we introduce small-angle x-ray diffraction as a new quantitative method to measure the degree of myofibrillar disorientation in myocardium. X-ray diffraction-based methods have several important advantages over other imaging techniques. X-ray diffraction information may be obtained from permeabilized tissue under near-physiological conditions at a known sarcomere length. It is difficult to control sarcomere length precisely in histological preparations because of the way that they are made. The observed x-ray diffraction patterns are the superposition of the individual diffraction patterns from all myofibrils exposed to the x-ray beam, with the azimuthal angular spread of the pattern providing an objective measure of the degree of departure from perfect angular alignment of myofibrils, i.e. "myofibrillar disorientation." In addition, x-ray diffraction patterns can be taken at the same time as mechanical measurements providing simultaneous structural and physiological information in real time. Both skinned and intact preparations can be studied with no change in procedures, but investigators should be cautioned against comparing results from skinned muscles with those from intact muscle because of the expected differences between the preparations, in particular the larger interfilament lattice spacings in skinned muscle.

Histopathological-based approaches have the advantage of using widely available microscopic tools and well-understood sample preparation protocols. These protocols, however, require multiple steps, including fixation, embedding and microtomy, staining, and image processing, each of which can introduce artifacts (52). None of these preparatory steps are necessary for x-ray diffraction. Furthermore, histological assessments only directly measure myocyte-level disarray and can only indirectly provide inferences into myofibrillar- and myofilament-level disarray. In this study, we have shown that myofibrillar orientation is sensitive to sarcomere length, and sample shrinkage during any of the histological processing steps may affect the accuracy of any measurement. Furthermore, with histological methods, only information encompassed within small regions of the sample selected for imaging and then for analysis is assessed, potentially leading to sampling bias. Histological methods are also exquisitely sensitive to the choice of the region identified. Regions must contain longitudinally oriented fibers, be free of histological artifacts, and void of fibrosis and spaces within the tissue. Although these criteria can be addressed in animal studies by simply examining more samples, satisfying all criteria may be difficult with histological sections in situations where sample material is limiting. Finally, if inappropriate criteria are used for image selection, image processing-based methods may result in a false-positive finding of myocyte disarray, as demonstrated in Fig. 6. Given these considerations, histological assessment of myocyte array requires a skilled operator well versed in fiber architecture.

Nonetheless, the principal advantage of histological methods is that they can be done anywhere with suitable microscopy facilities, whereas small-angle x-ray diffraction requires the use of a small-angle diffraction instrument on a synchrotron x-ray beamline. There are only a small number of such instruments worldwide. In the United States, only the BioCAT beamline 18ID at the Advanced Photon Source routinely does muscle diffraction, but the measurements described in this article would be feasible on other synchrotron x-ray beamlines worldwide. The ability to obtain small-angle x-ray diffraction patterns from frozen, biopsy-size pieces of human myocardium vastly expands the potential application of this method to assess the contributions of structure and physiology to human HF. This provides a motivation for expanding access to synchrotron facilities for studies of human cardiomyopathies, HFrEF, and HF with preserved ejection fraction.

Our proposed metric for characterizing myocardium provides otherwise inaccessible information at the myofibril/myofilament level in addition to that from the arsenal of techniques available for phenotypic analysis of diseased myocardium. We anticipate that this will be primarily useful for structurally charactering disease phenotypes and to generate hypotheses that can be tested experimentally to provide biophysical insights into disease etiology. Because this information is available in virtually all x-ray diffraction patterns from muscle, this can be done routinely as part of the analysis of every x-ray diffraction experiment where one is also acquiring lattice spacing and equatorial intensity ratio. X-ray diffraction assessment of myofibrillar orientation and myofilament disarray is seen as a complementary tool rather than a replacement for more accessible histological and molecular approaches, as well as functional assays that are routinely for characterization cardiomyopathies.

CONCLUSIONS

Small-angle x-ray diffraction of permeabilized myocardium can be used as a novel approach to precisely quantify myofibrillar orientation and the distribution of lattice spacings in permeabilized cardiac tissue under near-physiological conditions. These metrics can be used to precisely and objectively phenotypically characterize both human biopsies from diseased hearts and experimental systems, including transgenic animal disease models, as well as provide biophysical insights into disease etiology. The ability to obtain

full 2D x-ray patterns from frozen human myocardium opens up new translational opportunities to relate sarcomere structure to function in health and disease.

SUPPORTING MATERIAL

Supporting material can be found online at https://doi.org/10.1016/j.bpj.2022.01.009.

AUTHOR CONTRIBUTIONS

W.M., V.J., H.G., K.H.L., M.P., and M.L.-V. designed and performed research. W.M. and V.J. analyzed the data. W.M., V.J., J.R.P., M.I.A., A.C., and T.I. wrote the manuscript.

ACKNOWLEDGMENTS

This research was supported by NIH grants T32 GM73009 (to V.J), T32 HL007227 (to M.I.A.), HL128683 (to J.R.P), and R01HL139883 (to K.H.L) and the American Heart Association grants 19IPLOI34770173 (to T.I.), 2021AHAPRE216237 (to M.L.-V), and 111POST7210031 (to M.P.). This research used resources of the Advanced Photon Source, a U.S. Department of Energy (DOE) Office of Science User Facility operated for the DOE Office of Science by Argonne National Laboratory under Contract No. DE-AC02-06CH11357. This project was supported by grants P41 GM103622 and P30 GM138395 from the National Institute of General Medical Sciences of the National Institutes of Health. The content is solely the responsibility of the authors and does not necessarily reflect the official views of the National Institute of General Medical Sciences or the National Institutes of Health.

REFERENCES

1. Ait-Mou, Y., K. Hsu, …, P. P. de Tombe. 2016. Titin strain contributes to the Frank-Starling law of the heart by structural rearrangements of both thin- and thick-filament proteins. *Proc. Natl. Acad. Sci. U S A.* 113:2306–2311.
2. Caremani, M., F. Pinzauti, …, G. Piazzesi. 2019. Inotropic interventions do not change the resting state of myosin motors during cardiac diastole. *J. Gen. Physiol.* 151:53–65.
3. Brunello, E., L. Fusi, …, M. Irving. 2020. Myosin filament-based regulation of the dynamics of contraction in heart muscle. *Proc. Natl. Acad. Sci. U S A.* 117:8177–8186.
4. Madan, A., M. C. Viswanathan, …, A. Cammarato. 2020. TNNT2 mutations in the tropomyosin binding region of TNT1 disrupt its role in contractile inhibition and stimulate cardiac dysfunction. *Proc. Natl. Acad. Sci. U S A.* 117:18822–18831.
5. Viswanathan, M. C., W. Schmidt, …, A. Cammarato. 2020. A role for actin flexibility in thin filament-mediated contractile regulation and myopathy. *Nat. Commun.* 11:2417.
6. Gonzalez-Martinez, D., J. R. Johnston, …, J. R. Pinto. 2018. Structural and functional impact of troponin C-mediated Ca(2+) sensitization on myofilament lattice spacing and cross-bridge mechanics in mouse cardiac muscle. *J. Mol. Cell. Cardiol.* 123:26–37.
7. Varnava, A. M., P. M. Elliott, …, W. J. McKenna. 2001. Relation between myocyte disarray and outcome in hypertrophic cardiomyopathy. *Am. J. Cardiol.* 88:275–279.
8. Novo Matos, J., P. Garcia-Canadilla, …, V. Luis Fuentes. 2020. Micro-computed tomography (micro-CT) for the assessment of myocardial disarray, fibrosis and ventricular mass in a feline model of hypertrophic cardiomyopathy. *Sci. Rep.* 10:20169.
9. Wolf, C. M. 2019. Hypertrophic cardiomyopathy: genetics and clinical perspectives. *Cardiovasc. Diagn. Ther.* 9:S388–S415.
10. Marian, A. J., and E. Braunwald. 2017. Hypertrophic cardiomyopathy: genetics, pathogenesis, clinical manifestations, diagnosis, and therapy. *Circ. Res.* 121:749–770.
11. Seferovic, P. M., M. M. Polovina, and A. J. S. Coats. 2019. Heart failure in dilated non-ischaemic cardiomyopathy. *Eur. Heart J. Suppl.* 21:M40–M43.
12. Mosterd, A., and A. W. Hoes. 2007. Clinical epidemiology of heart failure. *Heart.* 93:1137–1146.
13. Murphy, S. P., N. E. Ibrahim, and J. L. Januzzi, Jr. 2020. Heart failure with reduced ejection fraction: a review. *JAMA.* 324:488–504.
14. Kirk, J. A., K. Chakir, …, D. A. Kass. 2015. Pacemaker-induced transient asynchrony suppresses heart failure progression. *Sci. Transl. Med.* 7:319ra207.
15. Ma, W., M. Childers, …, M. Regnier. 2020. Myosin dynamics during relaxation in mouse soleus muscle and modulation by 2'-deoxy-ATP. *J. Physiol.* 598:5165–5182.
16. Ma, W., H. Gong, and T. Irving. 2018. Myosin head configurations in resting and contracting murine skeletal muscle. *Int. J. Mol. Sci.* 19:2643.
17. Haselgrove, J. C., and H. E. Huxley. 1973. X-ray evidence for radial cross-bridge movement and for the sliding filament model in actively contracting skeletal muscle. *J. Mol. Biol.* 77:549–568.
18. Matsubara, I. 1980. X-ray diffraction studies of the heart. *Annu. Rev. Biophys. Bioeng.* 9:81–105.
19. Brenner, B., and L. C. Yu. 1985. Equatorial x-ray diffraction from single skinned rabbit psoas fibers at various degrees of activation. Changes in intensities and lattice spacing. *Biophys. J.* 48:829–834.
20. Shirai, M., D. O. Schwenke, …, J. T. Pearson. 2013. Synchrotron radiation imaging for advancing our understanding of cardiovascular function. *Circ. Res.* 112:209–221.
21. Mollenhauer, J., M. Aurich, …, T. C. Irving. 2003. X-ray diffraction of the molecular substructure of human articular cartilage. *Connect. Tissue Res.* 44:201–207.
22. Purslow, P. P., T. J. Wess, and D. W. Hukins. 1998. Collagen orientation and molecular spacing during creep and stress-relaxation in soft connective tissues. *J. Exp. Biol.* 201:135–142.
23. Geisterfer-Lowrance, A. A., S. Kass, …, J. G. Seidman. 1990. A molecular basis for familial hypertrophic cardiomyopathy: a beta cardiac myosin heavy chain gene missense mutation. *Cell.* 62:999–1006.
24. Green, E. M., H. Wakimoto, …, C. E. Seidman. 2016. A small-molecule inhibitor of sarcomere contractility suppresses hypertrophic cardiomyopathy in mice. *Science.* 351:617–621.
25. Anderson, R. L., D. V. Trivedi, …, J. A. Spudich. 2018. Deciphering the super relaxed state of human beta-cardiac myosin and the mode of action of mavacamten from myosin molecules to muscle fibers. *Proc. Natl. Acad. Sci. U S A.* 115:E8143–E8152.
26. Chen, C. Y., M. A. Caporizzo, …, B. L. Prosser. 2018. Suppression of detyrosinated microtubules improves cardiomyocyte function in human heart failure. *Nat. Med.* 24:1225–1233.
27. Aslam, M. I., V. S. Hahn, …, D. A. Kass. 2021. Reduced right ventricular sarcomere contractility in heart failure with preserved ejection fraction and severe obesity. *Circulation.* 143:965–967.
28. Blair, C. A., E. A. Brundage, …, K. S. Campbell. 2020. Heart failure in humans reduces contractile force in myocardium from both ventricles. *JACC Basic Transl. Sci.* 5:786–798.
29. Kawana, M., S. S. Sarkar, …, J. A. Spudich. 2017. Biophysical properties of human beta-cardiac myosin with converter mutations that cause hypertrophic cardiomyopathy. *Sci. Adv.* 3:e1601959.
30. Fischetti, R., S. Stepanov, …, G. B. Bunker. 2004. The BioCAT undulator beamline 18ID: a facility for biological non-crystalline diffraction and X-ray absorption spectroscopy at the advanced photon source. *J. Synchrotron Radiat.* 11:399–405.

31. Jiratrakanvong, J., J. Shao, …, T. Irving. 2018. MuscleX: software suite for diffraction X-ray imaging V1.13.1. https://doi.org/10.5281/zenodo.1195050.
32. Yu, L. C., A. C. Steven, G. R. Naylor, R. C. Gamble, and R. J. Podolsky. 1985. Distribution of mass in relaxed frog skeletal muscle and its redistribution upon activation. *Biophys J.* 47:311–321. https://doi.org/10.1016/S0006-3495(85)83921-7.
33. Granzier, H. L., and T. C. Irving. 1995. Passive tension in cardiac muscle: contribution of collagen, titin, microtubules, and intermediate filaments. *Biophys. J.* 68:1027–1044.
34. Fukuda, N., Y. Wu, …, H. Granzier. 2005. Titin-based modulation of active tension and interfilament lattice spacing in skinned rat cardiac muscle. *Pflugers Arch.* 449:449–457.
35. Granger, B. L., and E. Lazarides. 1979. Desmin and vimentin coexist at the periphery of the myofibril Z disc. *Cell.* 18:1053–1063.
36. Paulin, D., and Z. Li. 2004. Desmin: a major intermediate filament protein essential for the structural integrity and function of muscle. *Exp. Cell Res.* 301:1–7.
37. Wang, K., and R. Ramirez-Mitchell. 1983. A network of transverse and longitudinal intermediate filaments is associated with sarcomeres of adult vertebrate skeletal muscle. *J. Cell Biol.* 96:562–570.
38. Bloch, R. J., and H. Gonzalez-Serratos. 2003. Lateral force transmission across costameres in skeletal muscle. *Exerc. Sport Sci. Rev.* 31:73–78.
39. Danowski, B. A., K. Imanaka-Yoshida, …, J. W. Sanger. 1992. Costameres are sites of force transmission to the substratum in adult rat cardiomyocytes. *J. Cell Biol.* 118:1411–1420.
40. Sheng, J. J., H. Z. Feng, …, J. P. Jin. 2016. Increases of desmin and alpha-actinin in mouse cardiac myofibrils as a response to diastolic dysfunction. *J. Mol. Cell. Cardiol.* 99:218–229.
41. Capetanaki, Y., R. J. Bloch, …, S. Psarras. 2007. Muscle intermediate filaments and their links to membranes and membranous organelles. *Exp. Cell Res.* 313:2063–2076.
42. Pitoulis, F. G., and C. M. Terracciano. 2020. Heart plasticity in response to pressure- and volume-overload: a review of findings in compensated and decompensated phenotypes. *Front. Physiol.* 11:92.
43. de Tombe, P. P. 1998. Altered contractile function in heart failure. *Cardiovasc. Res.* 37:367–380.
44. Burchfield, J. S., M. Xie, and J. A. Hill. 2013. Pathological ventricular remodeling: mechanisms: part 1 of 2. *Circulation.* 128:388–400.
45. Maron, B. J., N. Sato, …, R. S. Chandra. 1979. Quantitative analysis of cardiac muscle cell disorganization in the ventricular septum. Comparison of fetuses and infants with and without congenital heart disease and patients with hypertrophic cardiomyopathy. *Circulation.* 60:685–696.
46. Davies, M. J., and W. J. McKenna. 1995. Hypertrophic cardiomyopathy–pathology and pathogenesis. *Histopathology.* 26:493–500.
47. Stewart, M. A., K. Franks-Skiba, …, R. Cooke. 2010. Myosin ATP turnover rate is a mechanism involved in thermogenesis in resting skeletal muscle fibers. *Proc. Natl. Acad. Sci. U S A.* 107:430–435.
48. Ma, W., M. Henze, …, T. Irving. 2021. The super-relaxed state and length dependent activation in porcine myocardium. *Circ. Res.* 129:617–630.
49. Spudich, J. A. 2019. Three perspectives on the molecular basis of hypercontractility caused by hypertrophic cardiomyopathy mutations. *Pflugers Arch.* 471:701–717.
50. Irving, T., and R. Craig. 2019. Getting into the thick (and thin) of it. *J. Gen. Physiol.* 151:610–613.
51. Jani, V., I. Aslam, …, S. Hsu. 2021. RV sarcomeres from LV-HFrEF patients with low PAPi have abnormal RV thick filament structure. *Circ. Res.* 129:AP505.
52. Taqi, S. A., S. A. Sami, …, S. A. Zaki. 2018. A review of artifacts in histopathology. *J. Oral Maxillofac. Pathol.* 22:279.

Biophysical Journal, Volume *121*

Supplemental information

Myofibril orientation as a metric for characterizing heart disease

Weikang Ma, Henry Gong, Vivek Jani, Kyoung Hwan Lee, Maicon Landim-Vieira, Maria Papadaki, Jose R. Pinto, M. Imran Aslam, Anthony Cammarato, and Thomas Irving

Supplemental Methods

X-ray Data Analysis. The data were analyzed using data reduction programs from the MuscleX software package developed at BioCAT (1). The angular divergence of the 1,0 equatorial X-ray reflections was measured by the "Scanning Diffraction" routine in the MuscleX software package. Briefly, the routine obtains 2D and 1D radially integrated intensities of the pattern. The 1D radially integrated intensity trace (red box in Fig S1) was fit assuming Gaussian profiles as a function of radial spacing for the diffraction peaks to calculate the standard deviation (width σ) of the peak intensity distribution in the radial direction. In this process, the routine obtains the integrated intensity of each equatorial reflection as a function of the integration angle (white box in Fig S1). Gaussian profiles (Fig 1D) are fit to the projected peak intensities to find the standard deviation of the orientation angle (angle σ) to calculate the angular divergence.

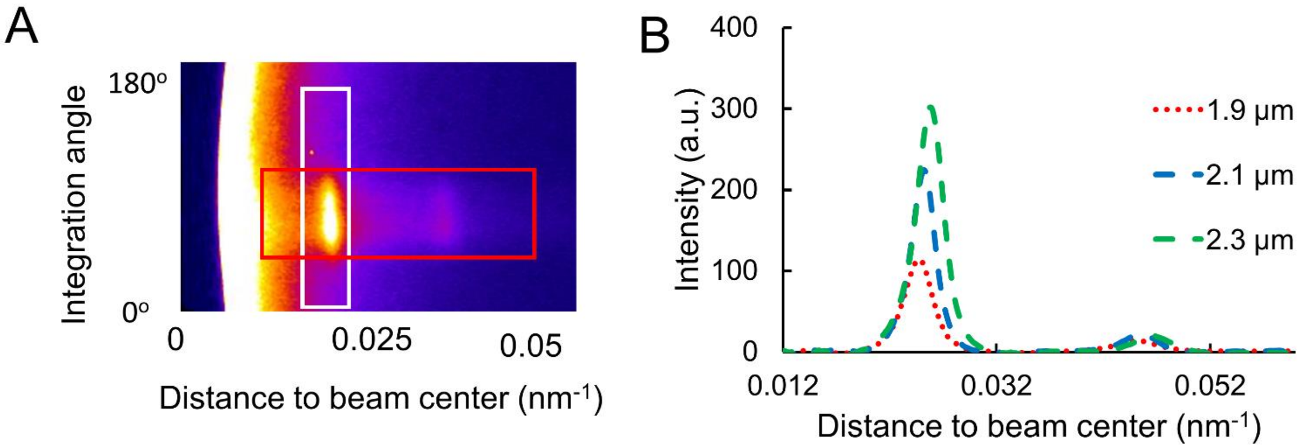

Figure S1. Determination of width σ: (**A**) Radially integrated X-ray pattern. White box: Integrates area of 1,0 reflection as a function of integration angle. Red box: Integrates area of 1,0 reflection as a function of the distance to beam center. (**B**) Radially integrated 1D intensity trace of 1,0 reflection (red box)

Sample Preparation for Electron Microscopy. Cardiac muscle fibers (< 2mm diameter) were excised from fresh or frozen pig heart ventricular wall and pinned down onto a Sylgard™ substrate under slight tension. Fibers were fixed with 2.5% glutaraldehyde in 0.1M cacodylate buffer pH 7.2 for 2 hours at room temperature followed by continued fixation overnight at 4 °C. Samples were then rinsed in cacodylate buffer three times for 20 minutes each at 4°C. The fibers were shipped overnight on ice from to the University of Massachusetts Medical School. The fibers were cut into small pieces and post-fixed with1% w/v OsO_4 in distilled water, then dehydrated in a series of graded ethanol solutions and embedded in Epon. Sections (70 nm thick) were stained with uranyl acetate followed by lead citrate as described (1). The samples are examined with an FEI Tecnai G2 Spirit electron microscope at 120 kV. Images were collected on a 4k X 4k CMOS camera (Gatan Rio 9). The A-band, Z-line, and sarcomere length were measured using ImageJ software (NIH).

Image Analysis of Electron Micrographs. Surrogates for width and angle sigma measured by X-ray diffraction were determined from power spectra from low magnification (8200 x) electron microscopy images (Figure S2 A). Briefly, two dimensional fast Fourier transforms, and magnitudes were calculated to generate a 2D power spectrum (Figure S2 B). The resulting magnitude images were rearranged such that the zero-frequency component is positioned at the center of the array. The equator was manually determined, and the power spectra were rotated such that the equator was horizontal. The intensity corresponding to the (1,0) intensity was manually selected for analysis. Numerical radial and angular integrals were quantified for the selected analysis region with 0.01-degree resolution and 0.01-pixel resolution, respectively. Following numerical integration, profiles were normalized to have an integral of one and fit to a univariate Gaussian, and variances were acquired. Width sigma was the variance of the Gaussian fit to the radial integral profile, while angle sigma was the variance of the Gaussian fit to the angular integral profile. The algorithm described was implemented in MATLAB R2018b (Mathworks), with code made available from the authors on request. An example output of the code is shown in **Figure S2**. Finally, for each EM image, sarcomere length was determined using ImageJ from at least 20 sarcomeres in each image and the scale bars provided with the micrographs. The pixel-sarcomere length calibration was also used to quantify the distance from the center of the (1,0) intensity from the power spectra, which corresponds to inter-myofibrillar spacing.

Supplemental Results

Eight individual thin section electron micrographs from porcine myocardium samples with an average sarcomere length of ~2 µm and fifteen individual electron micrographs from samples with an average sarcomere length of ~2.3 µm, each containing an average of 13.3 (range 9-20) myofibrils, were selected and analyzed using the power spectrum analysis procedure as described in the supplemental methods. Figure S2 A shows one such electron micrograph from a thin section containing ~12 myofibrils. Note that the long axes of the myofibrils are roughly, but not perfectly parallel to each other so that the long axes of the myofibrils have a distribution of angles around the average long axis. It is the width of this distribution (Angle Sigma) that is meant by myofibrillar disorientation. Table S1 shows the results of the results of the analysis of the power spectra of the electron microscopy images as described in the supplemental methods. There is a significant decrease ($p = 0.005$) in angle sigma (myofibrillar disorientation) when sarcomere length increases from 2 to 2.3 µm, qualitatively and quantitatively similar to corroborating the results of the X-ray diffraction studies of mouse myocardium shown in Figure 2A. During stretch, the distance between adjacent myofibrils increased slightly but significantly ($p=0.049$) and the width of the distribution of these inter-myofibrillar spacings appeared to increase but this difference was not significant ($p=0.083$), possibly because of the noise in the data (Figure S2 D). Note that this measurement is not directly analogous to the width sigma measured by X-ray diffraction which is due, at least partly, to the width of the distribution of *inter-myofilament* lattice spacings.

In the X-ray diffraction measurements, the sample volume in the X-ray beam is ~250 x 250 x 250 µm or ~ 1.6×10^7 µm^3 and the sample is translated during the exposure by one to several mm increasing the sample volume to at ~ 6×10^7 µm^3 or more. In the electron micrographs, the sample volumes in each micrograph are 70 nm x 15 x 15 µm or 17 µm^3, many orders of magnitude less than the X-ray diffraction volume. it would, therefore, be highly impractical to

sample sufficient numbers of longitudinal EM sections to be statistically equivalent to the information contained in a single X-ray pattern. The sample volumes in the histological sections, on average 8. X 10^7 μm^3, are much larger than in the electron micrographs. However, individual myofibrils are not visible in these micrographs and the number of myocytes typically visible in a histological preparation are of the same order as the number of myofibrils visible in a thin section electron micrograph. Therefore, a similar number of independent structures (10-20) are being interrogated in individual sections with both the histological and electron microscopy measurements, much less than the many thousands of myofibrils interrogated in the X-ray measurements.

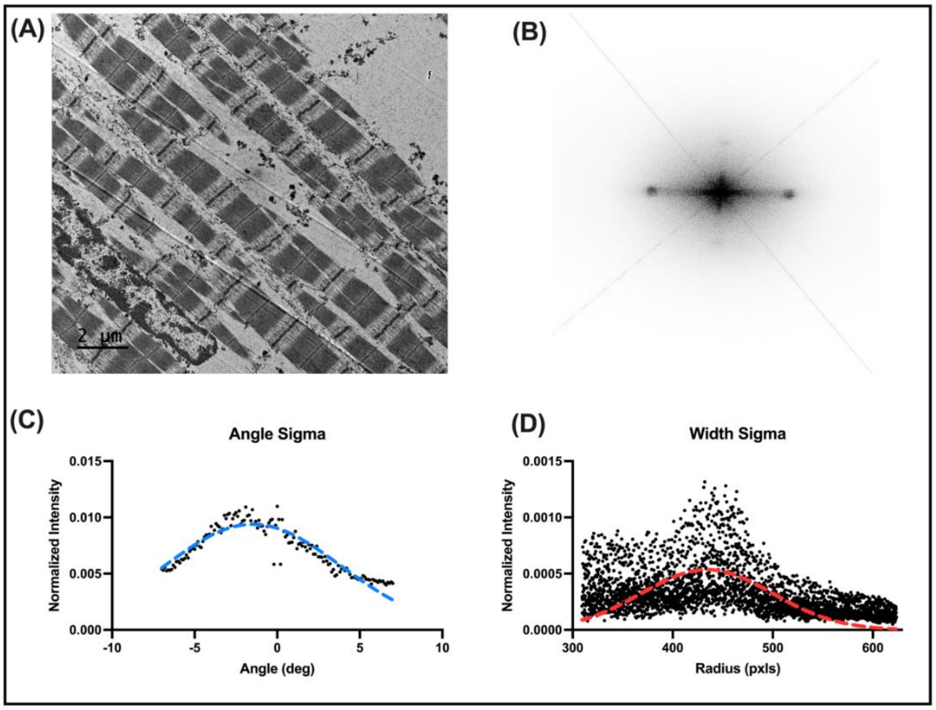

Figure S2. EM Image Analysis Workflow. (A) Representative EM image. (B) Power Spectrum. Spectrum was manually rotated to have the equator aligned horizontally. (C) Gaussian fit (blue) to quantify angle sigma of the angular integral of the (1,0) intensity from the power spectrum. (D) Gaussian fit (blue) to quantify width sigma from the radial integral of the (1,0) intensity from the power spectrum.

Table S1

**Sarcomere Length (μm)**	**Inter-myofibrillar Spacing (μm)**	**Angle Sigma (rad)**	**Width Sigma (μm)**
1.97 ± 0.012 (8)	1.70 ± 0.026 (8)	0.180 ± 0.038 (8)	0.276 ±0.044 (8)
2.30 ± 0.021 (15)	1.79 ± 0.024 (15)	0.142 ± 0.003 (15)	0.347 ±0.020 (15)

Supplemental References

1. Reynolds ES (1963) The use of lead citrate at high pH as an electron-opaque stain in electron microscopy. J Cell Biol 17:208-212
2. Jiratrakanvong, J., J. Shao, M. Menendez, X. Li, J. Li, W. Ma, G. Agam, and T. Irving. 2018. MuscleX: software suite for diffraction X-ray imaging V1.13.1. doi:10.5281/zenodo.1195050.
